# Coaching and guidance with patient decision aids: A review of theoretical and empirical evidence

**DOI:** 10.1186/1472-6947-13-S2-S11

**Published:** 2013-11-29

**Authors:** Dawn Stacey, Jennifer Kryworuchko, Jeff Belkora, B Joyce Davison, Marie-Anne Durand, Karen B Eden, Aubri S Hoffman, Mirjam Koerner, France Légaré, Marie-Chantal Loiselle, Richard L Street

**Affiliations:** 1 School of Nursing, University of Ottawa and Clinical Epidemiology Program, Ottawa Hospital Research Institute, 451 Smyth Road (RGN Room 1118), Ottawa, Ontario, K1H 8M5, Canada; 2 College of Nursing, University of Saskatchewan, 107 Wiggins Road, Saskatoon, Saskatchewan S7N 5E5, Canada; 3 Institute for Health Policy Studies, University of California, San Francisco, 3333 California Street, Suite 265, San Francisco, California 94118, USA; 4 College of Nursing, University of Saskatchewan, 107 Wiggins Road, Saskatoon, Saskatchewan S7N 5E5, Canada; 5 Department of Psychology, School of Life and Medical Sciences, University of Hertfordshire, College Lane Campus, Hatfield, AL 109AB, UK; 6 Department of Medical Informatics and Clinical Epidemiology, Oregon Health and Science University, 3181 SW Sam Jackson Park Road, Portland, Oregon 97239-3098, USA; 7 The Dartmouth Institute for Health Policy and Clinical Practice, Geisel School of Medicine at Dartmouth College, 46 Centerra Parkway (HB7250), Lebanon, New Hampshire 03766, USA; 8 Department of Medical Psychology and Medical Sociology, University of Freiburg, Hebelstr. 29, 79104 Freiburg, Germany; 9 Department of Family Medicine and Emergency Medicine, Université Laval, Pavillon Ferdinand-Vandry, 1050, avenue de la Médecine, Local 4617, Quebec, Province of Quebec G1V 0A6, Canada; 10 School of Nursing, Faculty of Medicine and Health Sciences, University of Sherbrooke, 150, place Charles-Le Moyne (Bureau 200), Longueuil, Province of Quebec J4K 0A8, Canada; 11 Department of Communication, Texas A&M University, College Station, Texas 77843-4234, USA

## Abstract

**Background:**

Coaching and guidance are structured approaches that can be used within or alongside patient decision aids (PtDAs) to facilitate the process of decision making. Coaching is provided by an individual, and guidance is embedded within the decision support materials. The purpose of this paper is to: a) present updated definitions of the concepts “coaching” and “guidance”; b) present an updated summary of current theoretical and empirical insights into the roles played by coaching/guidance in the context of PtDAs; and c) highlight emerging issues and research opportunities in this aspect of PtDA design.

**Methods:**

We identified literature published since 2003 on shared decision making theoretical frameworks inclusive of coaching or guidance. We also conducted a sub-analysis of randomized controlled trials included in the 2011 Cochrane Collaboration Review of PtDAs with search results updated to December 2010. The sub-analysis was conducted on the characteristics of coaching and/or guidance included in any trial of PtDAs and trials that allowed the impact of coaching and/or guidance with PtDA to be compared to another intervention or usual care.

**Results:**

Theoretical evidence continues to justify the use of coaching and/or guidance to better support patients in the process of thinking about a decision and in communicating their values/preferences with others. In 98 randomized controlled trials of PtDAs, 11 trials (11.2%) included coaching and 63 trials (64.3%) provided guidance. Compared to usual care, coaching provided alongside a PtDA improved knowledge and decreased mean costs. The impact on some other outcomes (e.g., participation in decision making, satisfaction, option chosen) was more variable, with some trials showing positive effects and other trials reporting no differences. For values-choice agreement, decisional conflict, adherence, and anxiety there were no differences between groups. None of these outcomes were worse when patients were exposed to decision coaching alongside a PtDA. No trials evaluated the effect of guidance provided within PtDAs.

**Conclusions:**

Theoretical evidence continues to justify the use of coaching and/or guidance to better support patients to participate in decision making. However, there are few randomized controlled trials that have compared the effectiveness of coaching used alongside PtDAs to PtDAs without coaching, and no trials have compared the PtDAs with guidance to those without guidance.

## Background

Coaching and guidance are structured approaches that can be used within or alongside patient decision aids (PtDAs). Both approaches are designed to help patients think about their options in preparation for discussing the decision with their practitioner(s). Underlying these concepts is the assumption that the process of decision making requires cognitive activities to understand options and their attributes, as well as two-way communication between the patient and his/her practitioner(s) to verify understanding of the options and clarify patients’ informed preferences. This decision-making process may also include significant others involved in the decision.

Coaching and guidance are important concepts in the field of PtDA design and development. In 2005, the International Patient Decision Aid Standards (IPDAS) Collaboration developed a set of evaluative criteria for assessing the quality of PtDAs [1]. As part of this process, the IPDAS Collaboration: a) identified coaching/guidance in deliberation and communication as one of twelve broad dimensions of PtDA design; b) proposed specific evaluative criteria for each of these twelve dimensions, including evaluative criteria for PtDA-based coaching/guidance; and c) invited consensus voters to indicate, within each dimension, the importance of each evaluative criterion. To help the consensus voters, the IPDAS Collaboration provided them with a twelve-chapter “background document” with information about the definitional, theoretical, and evidentiary background for each of the twelve dimensions, including a chapter specific to guidance and coaching [2]. One result of the consensus process was that the three evaluative criteria that focused on guidance (i.e., “PtDA provides a step-by-step way to make a decision”; “PtDA suggests ways to talk about the decision with practitioners”; and PtDA includes tools like worksheets or lists of questions to use when discussing options with a practitioner”) were considered highly important, in that each criterion was rated 8 out of 9 on an importance scale. Another result of the consensus process was that the two criteria that focused on coaching (i.e., “PtDA offers option of working with a trained coach to help patients consider the options” and “PtDA prepares one to talk about the decision with a practitioner”) were considered moderately important, in that both were rated 5 out of 9 [1].

The purpose of this paper is to move beyond the IPDAS Collaboration’s 2005 “background document” by: a) presenting updated definitions of the concepts *coaching* and *guidance*; b) presenting an updated summary of current theoretical and empirical insights into the roles played by coaching/guidance in the context of PtDAs; and c) highlighting emerging issues and research opportunities in this aspect of PtDA design.

## Coaching and guidance: definitions

*Coaching* is defined as the provision of support by a trained individual (either in person or remotely—for example by telephone or Internet), who is supportive but non-directive, for a patient or family facing a decision [3]. Using an iterative verbal exchange, elements of coaching include: a) assessing the patients’ decision-making needs; b) providing information on their options, benefits, and harms (e.g., verbally or with patient education resources such as PtDAs); c) verifying their understanding; d) clarifying their values associated with the attributes of the options, and their attitude toward risks; e) building their skills in deliberating, communicating, and accessing support; f) screening for barriers to implementation; and g) facilitating progress in decision making [4]. Although the patient may express their leaning toward a specific option to the decision coach, agreeing upon an option occurs during consultation with the practitioner rather than with the coach. Trained health professionals, students, or laypeople can provide coaching before and/or after using a PtDA, as part of its delivery, or in the absence of PtDAs. Coaching may also be referred to as decision support, counselling, navigation, and/or facilitation of the decision-making processes.

*Guidance* is defined as an explicit element embedded within the decision support materials that can facilitate a self-directed approach to the process of decision making. It can be included within the PtDA or as a resource used alongside the PtDA. Examples of elements used to provide guidance include: a) a list of steps or systematic approach for making a decision; b) a worksheet to help patients to clarify their values associated with the options’ attributes that can be shared with their practitioner; c) a list of questions and/or an invitation for users to identify questions to ask the practitioner (or decision coach); and/or d) an automated summary of the patients’ priorities and decisional needs (e.g., their knowledge, values, preference, the results of decision analysis) that can be given to the patient and shared with the practitioner(s), decision coach, and/or significant others involved in the decision.

## Theoretical justification

There are several rationales informing the use of coaching and guidance within or alongside PtDAs, some of which are from current or emerging decision-making theories or conceptual models [5]. In this section, we summarize the theoretical underpinnings of coaching and guidance from the IPDAS Collaboration’s original 2005 “background document” [2], and add new theoretical rationale that we identified.

### Achieving higher quality decisions

The objective of patient-oriented decision support is to help patients make higher quality decisions that are informed with the best available evidence and that reflect the patients’ values for the options’ attributes [6,7]. The main hypothesis underlying the use of guidance and coaching within or alongside PtDAs is that:

a) since patients are better able to participate in making decisions about their healthcare and to achieve a higher quality decision if they are supported in the process of thinking about a decision and discussing it with others,

and

b) since coaching and guidance may provide that support, by improving patients’ deliberation and communication skills, improving follow-through on a chosen option, and managing patients’ emotional distress,

then

c) coaching and guidance may help patients make higher quality decisions. See Table 1.

**Table 1:** Theoretical Rationale that Coaching and Guidance can Improve Decision Quality

By…	Coaching / Guidance can…
Increasing critical reflection, anticipating and avoiding common pitfalls (e.g., anchoring, misconceptions, etc.) that can undermine effective decision making; Taking someone through the steps of decision making; Helping patients become more informed by providing information, tailoring information, brainstorming and answering questions, stimulating patients to ask questions, and/or verifying understanding; Clarifying patients’ values by facilitating reflection, completing values clarification exercises, and/or sharing others’ experiences; and/or Building self-efficacy in decision making	Improve patients’ deliberation skills.

Helping patients prepare questions and identify concerns; Teaching skills for raising difficult subjects; Facilitating patients’ communicative capacity in the process of decision making; and/or Providing a worksheet or list of questions to share with the practitioner	Enhance patients’ skills in communicating with their practitioner(s).

Helping patients to anticipate and overcome barriers to implementing the desired option	Improve follow-through on the chosen option.

Helping patients to improve their ability to use coping skills; and/or Helping patients to enhance their problem-solving skills	Reduce patients’ emotional distress.

### Avoiding decision pitfalls

Patients and practitioners do not naturally follow the axioms of normative decision theory [8-10], but, when inconsistencies are highlighted, many willingly change their choices to be more aligned with these principles. Thus, explicit guidance or decision coaching in the steps of deliberation can overcome some of the common decision-making pitfalls.

### Improving the quality of patient-provider communication

Two-way communication is essential for shared decision making, but does not guarantee that shared decision making has occurred [11,12]. Two-way communication using high quality content (e.g., the provision and comprehension of evidence-based information, and the acknowledgment of individual values and preferences), coupled with strong patient-provider relationships, has been linked to greater satisfaction and positive health outcomes. Alternatively, poor communication has been linked to dissatisfaction, conflict, and worse outcomes [13]. Studies have documented the poor quality of communication between patients and providers [14,15]. Examples of poor communication include: a) one-way communication in which the physician dominates the discussion; b) a focus limited to medical facts, not thoughts/feelings or values associated with the options’ attributes; and c) documentation using a traditional problem-oriented note that does not incorporate elements of two-way communication or shared decision making [16]. Therefore, patients and practitioners may benefit from coaching and/or guidance to foster higher quality two-way communication.

### Enhancing learning

Consistent with principles of adult learning, patients learn in different ways [17-19]. Some patients prefer to learn from others, some prefer written, video, or interactive materials, and some prefer more than one approach to learning. Many researchers argue that learning and skill acquisition happen most effectively when patients are engaged in the process—often with the support of a coach—rather than by simply receiving factual information [17,20]. Patients are more apt to learn when messages and information are tailored to their situation, their needs, and their concerns [17,18,21].

### Managing emotional distress

A new diagnosis can cause significant emotional distress that often disrupts coping and problem-solving skills [22]. Although psychosocial services can help address excessive emotional distress, emotions are often important in personal decision making before, during, and after the decision [23]. First, emotions may propel the patient to deliberate and to act in support of or in opposition to an option. Second, emotions may give the patient positive or negative feedback. For example, during the decision process the patient may start to feel anxiety or fear about what is going to happen and may start anticipating decision regret. Decisional conflict is another type of emotional arousal that commonly occurs in patients making health decisions. It is defined as uncertainty about which course of action to take when choosing among actions that involve risk, loss, regret, or challenge to personal life values [24]. Some emotional arousal appears to be necessary to stimulate patients’ desire and capability to participate effectively in decision making [25]. The individualized approach used in coaching may improve the likelihood that patients’ emotions are considered throughout the decision-making process, particularly when clarifying the importance of attributes of options and acknowledging patients’ concerns.

### Decision making conceptual models that inform decision coaching

The Interprofessional Shared Decision Making Model, the Framework for Decision Coach Mediated Shared Decision Making, and the FAST model of critical reflection (defined below) have been used to inform the role of decision coaching provided alongside PtDAs (see Table 2) [3,26-29].

**Table 2:** Decision Making Conceptual Models to Inform Decision Coaching and/or Guidance

Conceptual Model	Goal	Provided by	SDM /Coaching or Guidance Process	Implemented and/or evaluated in
IP-SDM Model (coaching)	To assist two or more health professionals to achieve shared decision making with the patient	Health professional trained to support the patient’s involvement in SDM	1) Making explicit that a decision needs to be made, 2) Exchanging information (including the use of PtDAs), 3) Clarifying values/preferences, 4) Determining feasibility of options, 5) Reaching a choice, and 6) Implementing the chosen option.	Primary care (CA, US); Intensive care (CA, US); Nephrology (CA); Homecare (CA)

Framework for Decision Coach Mediated SDM (coaching)	To achieve higher quality decisions	Health professional	a) Assessing patients’ decisional conflict and related modifiable deficits in knowledge, values clarity and support; b) Tailoring decision support to meet patients’ needs by facilitating access to PtDAs and/or providing evidence-based information, verifying understanding, clarifying values, building skills in deliberation, communication and accessing support; c) Monitoring and facilitating patients’ progress in decision making; and d) Screening for factors influencing decision implementation, including patients’ motivation and self-efficacy, and other potential barriers impeding implementation.	Primary care call centre (CA, US, Chile); Cancer care (AU, UK, Japan); End of life care (CA); Various decisions in training of graduate students (CA)

FAST (coaching)	To improve participation in specialty or chronic care consultations	Students/trainees, peer navigators, allied health professionals	To help patients after they have reviewed a PtDA (or education materials in the absence of a PtDA) to formulate issues that they will subsequently analyze with their practitioner(s).	Orthopaedics (US, UK); Chronic care, (US, UK); Cancer care (US, UK)

Ottawa Decision Support Framework (guidance)	To address modifiable decisional needs contributing to decisional conflict	Incorporated as steps in PtDAs	Structures the process of decision making by making explicit a set of steps and encouraging patients to communicate their informed preferences with others involved in the decision (e.g., practitioner, family, friends)	Large variety of decisions (AU, CA, US, Japan, UK)

AU = Australia; CA = Canada; US = United States; UK = United Kingdom; SDM = shared decision making

The Interprofessional Shared Decision Making Model (IP-SDM Model) assumes that two or more healthcare professionals collaborate to achieve shared decision making with the patient either concurrently or sequentially; one of these professionals may undertake the decision coaching role. According to this model, the decision coach is a trained health professional. The interprofessional team members, including the decision coach, may have varying levels of involvement at different steps of the decision-making process, but overall they share a common understanding of this process (from deliberation to implementation of the chosen option). The IP-SDM model has been validated in primary care and home care clinical environments [28,30], and shown to be relevant in research studies evaluating patients’ decision making needs in the intensive care unit and renal dialysis decision making [31,32].

The Framework for Decision Coach Mediated Shared Decision Making expands the traditional patient-practitioner dyad to include the role of decision coaching, and integrates the Ottawa Decision Support Framework interventions as the key elements in the coaching role [3,33]. The Framework for Decision Coach Mediated Shared Decision Making assumes that higher quality decisions are achieved when patients and practitioners participate in decision making and a decision coach facilitates patient engagement in this process. The Ottawa Personal Decision Guide is a generic PtDA consistent with this framework that can be used to facilitate the provision of decision coaching with patients. Compared to controls, health professionals who were trained in decision coaching using PtDAs were more likely to assess patients’ decisional needs, discuss values associated with their options, and assess for support needed from others involved in the decision [34-36].

The FAST (Formulate issues, Analyze issues, Synthesize insights, Translate insights into action) model of critical reflection informed the decision coaching role as part of the process of PtDA implementation [26,37-40]. The coaching role in this program was designed to improve patient participation in consultations with their practitioner. Decision coaches include post-baccalaureate premedical students, nurses, and psychologists. Compared to usual care, men with prostate cancer randomized to the coaching intervention based on FAST had higher decision self-efficacy and lower decisional regret [40]. Another randomized controlled trial found that telephone delivery of coaching using the FAST model was as effective as in-person delivery of coaching, both producing pre/post improvements in decision self-efficacy [41].

### Decision making conceptual models that inform guidance

Decision making conceptual models that inform guidance for patients’ healthcare decision making are limited, to the best of our knowledge, to the Ottawa Decision Support Framework, which explicitly includes the element of guidance (see Table 2) [33]. This framework asserts that participants’ (e.g., individual, couple, family, practitioner) decisional needs will affect the achievement of a higher quality decision, which, in turn, affects actions or behaviours (e.g., delay), health outcomes, emotions (e.g., regret, blame), and appropriate use of health services [33,42]. Decision support interventions based on this framework are designed to address modifiable decisional needs. Guidance is one example of an explicit element included in decision support intervention (e.g., guiding clients to consider which benefits and harms are most important to them). The Ottawa Decision Support Framework has been commonly used a) for developing PtDAs for numerous decisions in Canada, Australia, the United States, Japan, and the United Kingdom [5,43], and b) for training healthcare professionals [44].

## Empirical evidence from studies of PtDAs

The following evidence summary for coaching/guidance is based on findings from the Cochrane Collaboration Review of PtDAs, which included randomized controlled trials published to the end of 2009 (N = 86) [43], as well as from an updated search of PtDA trials published to the end of 2010 (N = 12). For decision coaching, we also used a sub-analysis of trials that evaluated decision coaching within trials of PtDAs [4]. This sub-analysis a) included trials that allowed the impact of decision coaching provided by a healthcare professional to be compared to another intervention and/or usual care, and b) excluded trials in which patients were exposed to coaching in both arms of the trial [45-48]. One other trial was excluded because only 12 of 136 women (8.8%) in the intervention group were exposed to decision coaching [49]. In this single study in which decision coaching was optional, few women initiated contact with the coach or accepted coach-initiated contact. These authors surmised that women in this study may have opted out of coaching because they did not perceive, prospectively, any added value compared to just using the decision guide [49].

### Evidence about decision coaching

Of 98 trials of PtDAs, 11 (11.2%) included decision coaching provided by nurses, genetic counsellors, pharmacist, physicians (who were not the primary practitioner for the patient), psychologists, or health educators. Table 3 summarizes the findings from trials that evaluated decision coaching. A PtDA plus decision coaching compared to usual care improved knowledge and decreased mean costs [4]. Compared to baseline, both coaching alone and the PtDA alone improved knowledge, but there was no statistically significant difference in knowledge between groups. The impact of comparisons on other outcomes was more variable, with some trials showing positive effects (e.g., participation in decision making, satisfaction, actual choice) and other trials reporting no differences (e.g., participation in decision making, satisfaction, actual choice, values-choice agreement, decisional conflict, adherence, anxiety). Overall, none of these outcomes were worse when patients were exposed to decision coaching.

**Table 3:** Summary of Findings for Decision Coaching (“n” = number of studies)

	Positive Results *(p < 0.05)*	Mixed Results	No Difference
Coaching plus a PtDA versus Usual Care (n = 5)	- Improved knowledge [69-71] - Decreased mean costs [71,72] - Fewer physical limitations to lifestyle activities [72] - Decreased hysterectomies for more conservative options [72] - Increased psycho-education rather than medication for schizophrenia [69] - Increased single embryo transfers compared to double embryo transfer [71]	- Enhanced perceived/preferred involvement in decision making* [69,71] or no difference in participation [73] - Either more satisfied with the decision making process* [72] or no difference in satisfaction [73] - Improved feeling informed subscale* [71], but no difference in total decisional conflict [73]	- Values-choice agreement [70] - Satisfaction-uncertainty and control levels [71] - Anxiety or depression [71] - Uptake of genetic testing [70,73]

Coaching versus PtDA (n = 4)	- Increased values-choice agreement [74] - Similar improvements in knowledge [74-77] - Increased satisfaction with the decision making process [77]	- Decreased decisional conflict* [74] or no difference [75,77]	- Participation [75] - Preparation for decision making [75] - Use of hormones for menopause [74,75] or uptake of prenatal screening [77] - Adherence to hormones for menopause [74,75] - Anxiety or pregnancy outcomes [77]

Coaching plus a PtDA versus PtDA Alone (n = 4)	- Increased participation in decision making [78] - Decreased mean costs [72] - Similar improvements in knowledge [70]		- Values-choice agreement [70] - Satisfaction with the decision making process [72] - Uptake of hysterectomy [72], genetic testing [70], or prostate cancer screening [79]; - Health outcomes [72], anxiety or depression [78]

### Evidence about guidance

Among 98 randomized controlled trials, 63 (64.3%) used PtDAs that contained some sort of explicit guidance in deliberation and/or communication. Examples of PtDAs that did not provide explicit guidance used simple paper-based consent formats [50,51] or computer-based programs with conjoint analysis [52]. The amount of guidance varied considerably. Table 4 summarizes the types of guidance provided (these are not mutually exclusive). Only one trial of PtDAs compared guidance provided by different individuals [43]. In this trial, findings revealed that, compared to the same PtDA administered by a research assistant prior to the consultation, a PtDA administered by the physician during the consultation showed a non-statistically significant trend of higher acceptability of the PtDA and lower decisional conflict [53]. Given that more detailed PtDAs are more likely to include one or more of these elements of guidance in deliberation or communication, we also report evidence comparing simple to detailed PtDAs. Compared to simpler PtDAs, more detailed PtDAs produced higher gains in knowledge, more realistic expectations, and a greater match between patients’ values and their chosen option [43]. However, the link between detailed PtDAs, explicit guidance, and effectiveness needs to be tested empirically.

**Table 4:** Types and Frequency of Guidance Provided within PtDAs

Type of Guidance	Frequency of occurrence in published studies
Step-by-step process for making the decision	27

Worksheet with questions relevant to the decision-making process	31

Administered by the physician in the consultation or by a research assistant (e.g., decision boards, decision cards, or computer program)	9

Explicitly tells patients to communicate with their practitioners by asking questions and sharing their preferences	7

Interactive computer programs: inherently guided the patient through the PtDA and decision-making process	6

Summaries that could be shared with the practitioner(s) during the consultation (e.g., completed worksheets/workbook, computer printout indicating treatment preferences, letter with results of decision analysis)	42

For more information, see “Table of Characteristics of Included Studies” in the Cochrane Collaboration Review of Patient Decision Aids [43]

## Discussion

Our review of coaching and guidance focused on studies satisfying the inclusion criteria from the 2011 Cochrane Review of Patient Decision Aids. Our findings revealed that theoretical evidence continues to justify the use of coaching and/or guidance to better support patients to participate in decision making. However, there are few randomized controlled trials that have compared the effectiveness of coaching used alongside PtDAs to PtDAs without coaching, and no trials have compared the PtDAs with guidance to those without guidance. Below, we discuss these observations in more detail.

### Our findings about guidance

As noted above, we found no known randomized trials that have isolated and measured the effect of guidance in PtDAs and/or of summary tools used to inform the decision making process within the patient-practitioner consultation. Therefore, research is required to determine the contribution of guidance within PtDAs or used alongside PtDAs. Research is underway to better understand the constructs of automated guidance within technology-based decision support systems.

### Our findings about decision coaching

We were interested in comparing our findings from a systematic review of PtDAs that included studies of coaching alongside PtDAs to findings from other systematic reviews that did not include PtDAs. Therefore, we identified systematic reviews that included coaching as part of an intervention to enhance the quality of patient-physician communication [54,55].

One systematic review included coaching interventions such as engaging the patient in discussion of the problem, encouraging questions and participation in decision making about management, as well as discussion of emotions and feelings [54]. These interventions produced positive psychological outcomes in 26 of 35 trials (e.g., reduced anxiety and depression, enhanced quality of life, and well-being) and positive physical outcomes in 11 of 25 trials (e.g., reduced pain and improved functional status). Interestingly, PtDA studies with or without coaching focused more on educational and decision-related proximal outcomes than on psychological outcomes. Generally, PtDA studies have included some secondary psychological measures, such as anxiety, and found no difference. This is sometimes interpreted along the lines of “decision support does not psychologically harm patients”, which would presumably be occurring if it increased anxiety [43]. However, broader literature on coaching (outside of PtDAs) suggests psychological benefits such as reductions in anxiety [54]. Further research is needed to understand the impact of coaching delivered with PtDAs on psychological outcomes.

Another systematic review of interventions directed at enhancing patients’ participation in the consultation included interventions such as coaching that focused on question-asking, raising concerns, and requesting clarification or checking understanding [55]. Of 16 trials, 10 reported significant increases in patient participation in the consultation and 5 had nonsignificant increases. Furthermore, patients who had greater participation also experienced more sense of control and preferred to be more active in the consultation. These results suggest that coaching outside of the PtDA context produces similar benefits as those we found in our review for patient participation.

Although our findings indicated that coaching improved patients’ knowledge and showed no detrimental effect on other patient outcomes, little detail was reported on the “dose” of coaching used in these trials. And, in fact, in the single trial in which patients were given the option of coaching, few took advantage of this intervention [49].

Our findings also lack detail on population or system-level outcomes. A randomized controlled trial featuring coaching and PtDAs provides insight into the influence of coaching when implemented at a large scale with members of a health insurance plan [56]. This trial involved 174,120 individuals with selected medical conditions, and featured telephone-based coaching on topics such as shared decision making, self-care, and behavioural change [56]. Individuals were assigned to one of two groups, which were then randomized to usual versus enhanced outreach. Compared to usual outreach, the enhanced outreach group had lower cut points for offering coaching to individuals, based on their predicted future costs and health conditions, and these individuals received more outreach calls (five versus three calls). Findings revealed that patients in the enhanced outreach group were more likely to receive coaching (22.2% versus 6.3%) and be sent a PtDA (41.1 versus 11.4 per thousand per year) for preference-sensitive conditions that put them at risk for a surgical intervention (e.g., lumbar surgery, knee/hip replacement, cardiac revascularization, prostatectomy, hysterectomy). Consistent with findings from the Cochrane Review of Patient Decision Aids [43], the authors report that “the number of surgical procedures performed for the six targeted preference-sensitive conditions in either the inpatient or outpatient setting was 9.8% lower in the enhanced-support group than in the usual-support group (*p* = 0.04)”. This finding is interesting, in that it suggests that expanding the reach of coaching reduces healthcare resource utilization under pragmatic, large-scale conditions.

### Implications for research, policy, and practice

Several implications for research, policy, and practice have been highlighted by our updated review of the definitional, theoretical, and evidentiary basis for using coaching and guidance within or alongside PtDAs. We discuss these implications in the following text.

#### Clarifying the concepts

The original definitions for coaching and guidance that had been written for the IPDAS Collaboration’s 2005 “background document” were primarily based on concepts of health coaching [57,58] and communication processes [59-62]. Compared with those earlier attempts, we have provided more explicit definitions, in order to simplify how we communicate about those two major concepts. To update the definition of “coaching” (see **Coaching and guidance: definitions** – *coaching*), we removed the term “balanced instruction”, replacing it with “non-directive support”. As well, more details on the elements of coaching were added in order to be consistent with more recent literature on decision coaching [3,4,28,63,64]. To update the definition of “guidance” (**see Coaching and guidance: definitions** – *Guidance*), we created a more succinct definition and added the automated summary of the patients’ decisional information that is used in some clinical settings and that is available as a print-out for some online PtDAs [65,66].

Conceptual clarity has been somewhat impaired with the emergence of telephone menus or e-tools often called automated decision coaching [63]; however, human interaction is not involved and therefore it fits with our definition of guidance rather than coaching. To further enhance conceptual clarity for coaching and guidance, a concept analysis should be conducted.

#### New theoretical frameworks inclusive of coaching

Since the coaching and guidance chapter was written for the original 2005 IPDAS “background document”, there has been a theory analysis of existing shared decision making conceptual models [29], and several newer models have appeared in the literature that make explicit the role of coaching [3,26-28]. With renewed attention to coaching, barriers interfering with the delivery of decision coaching in routine clinical practice are important to consider. Examples of barriers include: a) lack of awareness, knowledge, and skills in decision coaching among health professionals; b) inadequate decision coach training; c) lack of time in clinical practice interfering with developing and using decision coaching skills; and d) inadequate environmental supports to facilitate the decision coach role [38,64,67].

Therefore, in order to better address barriers, it seems that the theoretical models underpinning decision coaching interventions need to be incorporated into broader conceptual frameworks about implementation. To date, theoretical work has contributed to understanding the components of interventions (PtDAs, coaching, and/or guidance) that produce outcomes; however, to better understand the potential for broader implementation of SDM approaches, next steps include gaining an understanding of: 1) the mechanisms or reasons why the interventions have the effects that they do; and 2) the ways in which the elements of context influence these mechanisms. For example, if coaching works because people feel empowered as legitimate decision makers, then such an intervention might be most effective in a policy environment that promoted patient involvement, but be least effective in an environment that reinforced the need to have physician agreement with the decision (an aspect of physician power). Using conceptual frameworks or theories to guide studies of coaching or guidance with PtDAs is essential to understanding how such interventions have impact.

#### Lack of empirical evidence to support coaching or guidance with PtDAs

More evaluative investigation is required to understand: a) the added effect of decision coaching beyond the PtDA; b) which population(s) could most benefit from decision coaching; c) who should deliver this intervention—a health professional or lay coaches; d) the effect of a coaching intervention that is tailored to the unique factors influencing patients’ baseline decisional needs and/or their decision-making process; and e) the effect of coaching or guidance alone. Furthermore, when decision coaching is provided by healthcare professionals within a clinical setting, can its delivery be spread out among different members of the interprofessional team, or does one member of the team need to take responsibility for this role? Another area requiring further evaluation is the use of decision coaching in patients with chronic conditions in which the decision situation is revisited over time and/or there is a series of different decisions to be made [68].

### Limitations

There are two main limitations to consider for this review. First, we did not systematically review the literature for theoretical work related to both concepts; rather we expanded the original theoretical rationale to include literature identified by the research team. Second, the effectiveness of coaching and guidance was limited to a sub-analysis of existing systematic reviews and, therefore, we did not synthesize the literature on the effect of coaching or guidance alone.

## Conclusions

Although there is theoretical evidence to support inclusion of coaching and guidance with PtDAs, there are few randomized controlled trials that have evaluated the effectiveness of coaching used alongside PtDAs and no trials that have evaluated the effectiveness of guidance. Findings may be used by researchers who are developing or evaluating PtDAs and by key stakeholders who are involved in implementing PtDAs within routine practice.

## List of abbreviations used

FAST: Formulate issues, Analyze issues, Synthesize insights, Translate; IP-SDM Model: Interprofessional Shared Decision Making Model; PtDAs: patient decision aids

## Competing interests

Jeff Belkora has received funding for partial salary support since 2004 and Jennifer Kryworuchko received a doctoral fellowship award from the Informed Medical Decisions Foundation, a not-for-profit (501 (c)3 ) private foundation (http://www.informedmedicaldecisions.org). The Foundation develops content for patient education programs. The Foundation has an arrangement with a for-profit company, Health Dialog, to co-produce these programs. The programs are used as part of the decision support and disease management services Health Dialog provides to consumers through health care organizations and employers.

Dawn Stacey, Karen Eden, Mirjam Korner, France Legare, Marie-Chantal Loiselle, Aubri Hoffman, Joyce Davison, Mary Anne Durand, and Richard Street have nothing to declare.

## Authors' contributions

DS led the group’s work on the update process. JK helped to draft the manuscript. All authors participated in the review process, provided content expertise, and read and approved the final manuscript.

## Declarations

Publication of this supplement was funded by an unrestricted grant from the Informed Medical Decisions Foundation and by the University Research Chair in Knowledge Translation to Patients, University of Ottawa, Canada. Administrative and editorial support for this supplement was provided by The University of Texas MD Anderson Cancer Center.

This article has been published as part of *BMC Medical Informatics and Decision Making* Volume 13 Supplement 2, 2013: The International Patient Decision Aid Standards (IPDAS) Collaboration’s Quality Dimensions: Theoretical Rationales, Current Evidence, and Emerging Issues. The full contents of the supplement are available online at http://www.biomedcentral.com/bmcmedinformdecismak/supplements/13/S2.
